# Gut microbiota in perioperative neurocognitive disorders: current evidence and future directions

**DOI:** 10.3389/fimmu.2023.1178691

**Published:** 2023-05-04

**Authors:** Yan Sun, Kexin Wang, Weiliang Zhao

**Affiliations:** Department of Anesthesiology, China-Japan Union Hospital of Jilin University, Changchun, Jilin, China

**Keywords:** perioperative neurocognitive disorders, gut microbiota, gut-brain axis, inflammation, immunity

## Abstract

Perioperative neurocognitive disorders (PND) is a common surgical anesthesia complication characterized by impairment of memory, attention, language understanding and social ability, which can lead to a decline in the quality of life of patients, prolong the hospitalization period and increase the mortality rate. PND has a high incidence rate, which has a great impact on postoperative recovery and quality of life of patients, and has caused a heavy economic burden to society and families. In recent years, PND has become an important public health problem. The high risk population of PND is more prone to gut microbiota imbalance, and gut microbiota may also affect the inflammatory response of the central nervous system through the microbiota-gut-brain axis. Meanwhile, Neuroinflammation and immune activation are important mechanisms of PND. Regulating gut microbiota through probiotics or fecal bacteria transplantation can significantly reduce neuroinflammation, reduce the abnormal activation of immune system and prevent the occurrence of PND. This review summarizes the research progress of gut microbiota and PND, providing basis for the prevention and treatment of PND.

## Introduction

Perioperative neurocognitive disorders (PND) is common and significant complication of surgery, affecting a significant proportion of patients, occurred in up to 50% of elderly patients undergoing major or high-risk operations (1, 2). PND is characterized by changes in cognitive function, including memory impairment, attention deficits, and reduced executive function, which can persist for months or years after surgery (3). Despite the growing awareness of PND and its impact on patients, its underlying mechanisms are still not fully understood. Studies demonstrated that PND was mainly associated with neuroinflammation, oxidative stress, the abnormal accumulation of β-amyloid protein, and the damage of neural synaptic function (4, 5). The latest research shows that gut microbiota can regulate the central nervous function through the microbiota-gut-brain axis (6, 7). Therefore, this article reviews the mechanism of gut microbiota disorder in PND, in order to help explore reasonable early treatment strategies.

## Dysbiosis of gut microbiota in patients with PND

The human gut is home of a complex and dynamic community of microorganisms, collectively known as the gut microbiome. This community plays a crucial role in human health, influencing various physiological processes, including immunity, metabolism, and brain function (8–10). Recent studies have suggested that the gut microbiome may play a role in the development of PND by disrupting the gut-brain axis, which is the communication pathway between the gut and the central nervous system (11, 12). The gut microbiome is known to be sensitive to changes in the environment, such as surgery and the administration of antibiotics, which can alter the composition and function of the gut microbiome (13, 14). This alteration in the gut microbiome has been associated with cognitive impairment and the development of PND (15). In animal models, gut microbiome alterations have been shown to result in neuroinflammation, oxidative stress, and changes in neurotransmitter levels, which are all factors known to contribute to cognitive impairment (16). In recent years, several studies have investigated the relationship between the gut microbiota and PND. A recent study found that the gut microbiota composition was altered in patients with PND (17). Another study found that preoperative use of antibiotics, which can alter the gut microbiome, was associated with an increased risk of PND (18).

## Role of the gut microbiota in the pathogenesis of PND

Under normal circumstances, the gut microbiota forms an ecological balance in the human body, maintaining the health of the human intestinal micro-ecology (19). In recent years, the research on the gut microbiota-gut-brain axis has made people realize that gut microbiota affects the brain not only through the neuroanatomical pathway, but also through the endocrine system, immune system and metabolic system (20, 21). The imbalance of gut microbiota will destroy the intestinal barrier, promote inflammatory factors and toxic metabolites to enter the blood circulation, and destroy the blood-brain barrier (22). It aggravates the inflammatory reaction and immune imbalance of the central nervous system, thus promoting the occurrence of PND (23). At the same time, surgery and anesthesia will stimulate gut microbiota disorder, and psychological diseases such as anxiety and depression will also induce gut microbiota disorder, which may be a risk factor for PND (24).

## Gut microbiota and central nervous inflammatory response

Inflammation of the central nervous system is the main pathological process of PND (25). The surface of human intestinal mucosa is closely arranged by a layer of monolayer cells to form a barrier, preventing harmful substances from entering the blood circulation and protecting the health of the host. In the same way, for the brain, the blood-brain barrier also protects the normal operation of the brain by maintaining its own integrity (26). A systematic review study showed that the gut microbiota of patients had significant changes after surgery, regardless of whether digestive system surgery was performed or not, and the proportion of *Gram-negative* bacteria increased (27). LPS in the cell wall of *Gram-negative* bacteria can lead to an increase in intestinal permeability (28). The damaged intestinal barrier can promote the entry of intestinal bacteria and intestinal toxic metabolites into the blood circulation, leading to a significant increase in pro-inflammatory factor production (29). These inflammatory mediators can activate the corresponding inflammatory signal pathway, and pass through the blood-brain barrier through the specific receptors and transporters on the endothelial cell surface of the blood-brain barrier to induce the activation of microglia and neuroinflammation in the brain (30). Meanwhile, these inflammatory mediators can reduce the expression of tight junction proteins such as occulin and claudin-5, destroy the integrity of blood-brain barrier, and enter the brain to activate adaptive immune cells, leading to brain immune instability (31). In animal experiments, injecting LPS into the abdominal cavity of mice can lead to learning and memory impairment by increasing the permeability of the blood-brain barrier, and the permeability of the blood-brain barrier will recover after rebuilding the balance of gut microbiota (32). It has been reported that after oral supplementation of intestinal prebiotics, the number of probiotics such as lactobacillus and bifidobacterium in the intestine of PND mice increased, the level of inflammatory factors in the hippocampus decreased, and the cognitive function improved (33).

## Gut microbiota and neurotransmitter

Since the discovery of the enteric nervous system in the 19th century, more and more studies have been carried out on the interaction between the gut and the brain. With the deepening of the research, the concept of the gut microbiota-gut-brain axis has been summarized (34). The brain communicates with the intestine through multiple parallel pathways, including two branches of the autonomic nerve, the hypothalamus-pituitary-adrenal axis and the sympathetic-adrenal axis, and the descending monoaminergic pathway (35). A previous study has shown that trimethylamine oxide, a metabolic derivative of gut microbiota, can mediate neuroinflammation and increase the production of reactive oxygen species in the hippocampus through microglia, thus increasing the susceptibility to oxidative stress induced by surgery and aggravating PND (36). Acetylcholine, 5-HT, dopamine, GABA and other neurotransmitters affect the function of the central nervous system through the central cholinergic and dopaminergic nerves (37). The decline of learning and memory ability is often accompanied by changes in the level of neurotransmitters in the relevant brain areas (38). Intestinal microorganisms can produce tryptophan, the precursor of 5-hydroxytryptamine, which can pass through the blood-brain barrier to produce 5-HT and affect many functions of the nervous system (39). Surgical anesthesia disturbs gut microbiota, destroys the synthesis and metabolic homeostasis of 5-HT involved in intestinal endocrine cells and intestinal flora, which can lead to an increase in the production of 5-HT in the body, thus affecting mood, behavior and postoperative gastrointestinal peristalsis (40). In addition, lactobacillus, bifidobacterium, streptococcus and other bacteria in the intestine participate in the process of glutamic acid metabolism and GABA synthesis (41). GABA synthesized by gut microbiota directly stimulates the secretion of 5-HT by intestinal chromaffin cells, and affects the level of brain-derived nutrients, dopamine, etc (42).

## Gut microbiota and Aβ protein

Age is an independent risk factor for PND (43). Neuron degeneration may exist in elderly patients before operation, which is manifested by accumulation of Aβ protein in the brain and τ protein hyperphosphorylation (44). Aβ protein from gut microbiota (produced by *Escherichia coli*, *Bacillus subtilis*, *Salmonella*, etc.) can enter the circulation through the damaged intestinal wall (45). Although the primary structure of enterogenous Aβ protein is different from that of brain Aβ protein, its tertiary structure is very similar. Therefore, enterogenous Aβ protein may trigger cross-immune response and trigger over-activation of pro-inflammatory signal pathway in brain. After surgical anesthesia, the increase in the proportion of E. coli in the intestine can promote the deposition of Aβ protein in and out of nerve cells, and can promote neuronal synaptic dysfunction and even lead to cell death by activating the reactive changes of glial cells around nerve cells. In addition, the deposition of Aβ protein interferes with the expression of NMDA receptor in hippocampal neurons and cortex, reduces synaptic plasticity, and leads to cognitive impairment. The increase of Aβ protein caused by gut microbiota disorder can be improved by fecal microbiota transplantation (FMT).

## Target gut microbiota for the treatment of PND

### Fecal microbiota transplantation

PND is accompanied by the occurrence of neuroinflammation and the disorder of gut microbiota during operation. Dysbiosis of gut microbiota will promote the progress of neuroinflammation, so regulation of gut microbiota will have a certain effect on the treatment of PND. Prebiotics, probiotics and fecal bacteria transplantation can affect gut microbiota and thus affect cognitive function (46). Fecal microbiota transplantation (FMT) is a new method to treat gut microbiota disorder in recent years. It is a proposed therapeutic strategy that aims to address the dysregulation of the gut microbiota. It is implemented by transplanting the microbiota of ideal donors to supplement or replace the gut microbiota of target recipients. FMT has been used for the treatment of many diseases, such as IBD, cancer, liver diseases, rheumatoid arthritis, etc (47, 48). Current research has proved that FMT can improve the cognitive function of patients with Parkinson’s disease and AD (49). A previous study found that probiotics and fecal bacteria transplantation could attenuate intestinal inflammation and hippocampal inflammation in PND model rats, suggesting that fecal bacteria transplantation and probiotics were effective in improving PND (50). In addition, some recent studies have evaluated the efficacy of fecal microbiota transplantation (FMT) in the treatment of cognitive impairment in patients with AD. Sun et al. showed that after FMT treatment of APPswe/PS1dE9 transgenic mice, their spatial learning ability was improved, and the aggregation of Aβ protein in their brain was reduced, suggesting that FMT could improve the cognitive function of AD patients (51).

### Probiotics and prebiotics

Probiotics can promote the growth and reproduction of beneficial microbiota, improve the balance of host intestinal microbiota, and play a positive role in the recovery of brain function through various physiological channels (52). It is found that the activity of specific functional areas in the brain of healthy adults who take probiotics for a long time is higher. Researchers from institutions such as Islamic Assad University have found for the first time that probiotics can improve the cognitive function of human brain (53), and some studies have also proved that probiotics can reduce the cognitive function damage of mice after surgery (54). Furthermore, probiotics can regulate gut microbiota, reduce neuroinflammation, and alleviate cognitive dysfunction related to neuroinflammation during cardiac surgery (55). Yang et al. showed that continuous use of prebiotics for 3 weeks before operation can effectively reduce the incidence of cognitive dysfunction after abdominal surgery in rats, and effectively inhibit the release of IL-6 and activation of microglia in the hippocampus (33).

### Dietary regulation

The composition and diversity of gut microbiota will change according to the dietary structure, and the production of corresponding metabolites will also be affected. Gut microbiota can synthesize a variety of essential vitamins, amino acids and fatty acids, participate in glucose and protein metabolism, and also regulate BDNF, synaptophysin, postsynaptic density protein and other nutritional factors that affect the development and plasticity of the nervous system. A high-fat or high-energy diet will increase the displacement of LPS, promote the development of inflammation and insulin resistance. The dietary fiber diet is conducive to the maintenance of gut microbiota diversity and the inhibition of inflammation. Furthermore, SCFA is the major metabolic product obtained from the decomposition and fermentation of dietary fiber in the gastrointestinal tract by intestinal microorganisms. The current research shows that SCFA may participate in the changes of people’s cognitive and neurological functions from aspects of immunity, neuroendocrine, and blood-brain barrier (56). Some research showed that butyrate, one of SCFA, can activate the secretion of BDNF and reduce neuroinflammation. Butyrate can also trigger the expression of glutathionease, thus alleviating the oxidative stress reaction (57). Therefore, supplementing dietary fiber to obtain more SCFA may be an effective measure to treat cognitive impairment.

## Conclusions

In conclusion, there have been many studies proving the correlation between gut microbiota and PND. Stress reaction during surgical anesthesia, gastrointestinal motility abnormalities caused by anesthetic drugs or muscle relaxants, and the use of antibiotics during perioperative period could increase the risk of gut microbiota disorder. However, the research on the direct relationship between gut microbiota and PND is relatively small, and more are phenomenological research and exploration, which is not deep enough. Future research can explore its mechanism by combining current genomics and other methods. Deeper exploration of whether there are specific flora and specific signal pathways, screening of relevant specific microorganisms, or finding out microorganisms that can improve cognitive dysfunction, may become the treatment target of PND.

## Author contributions

YS, KW, and WZ wrote the manuscript; WZ revised the review. All authors contributed to the article and approved the submitted version.
